# Horizon scanning at EMA: preparing for the medicines of the future

**DOI:** 10.3389/fmed.2026.1800213

**Published:** 2026-04-16

**Authors:** Patrícia Dias Correia, Valentina Cordo’, Oriane Blanquie, Enrico Tognana, Dimitrios-Ilias Balourdas, Constantinos Ziogas, Falk Ehmann

**Affiliations:** 1 Regulatory Science and Innovation Task Force, European Medicines Agency (EMA), Amsterdam, Netherlands; 2 Department of Dermatology, University Medical Center of the Johannes Gutenberg-University Mainz, Mainz, Germany; 3 Cellular Protein Chemistry, Bijvoet Centre for Biomolecular Research, Utrecht University, Utrecht, Netherlands

**Keywords:** emerging trends, EU innovation network, European medicines regulatory network, foresight, horizon scanning, innovation in medicines, medical technologies, medicines development

## Abstract

This article provides insights into the European Medicines Agency’s approach to horizon scanning as a foresight tool to support early identification, analysis and preparedness for emerging trends, technologies and challenges relevant to medicines development and authorization. Emerging topics are identified through a continuous scanning of several sources of information, including key trends identified by leading institutions such as the World Health Organization, the European Commission Joint Research Centre, and the European Innovation Council. This perspective sets out the scope, impact and outputs of horizon scanning within the European medicines regulatory network, which include topic-specific reports, collaborative workshops and webinars, and contributions to regulatory policy, strategic planning, capacity building and research prioritization. It also emphasizes the European medicines regulatory network’s commitment to collaboration and knowledge sharing across European and international organizations. Through these efforts, horizon scanning supports regulatory preparedness and resilience of the European medicines regulatory system.

## Introduction

The European medicines agencies network (EMAN), which includes national competent authorities (NCAs) and the European Medicines Agency (EMA), works under an overarching strategy that aims at “seizing opportunities in a changing medicines landscape” (1). The EMAN strategy (EMANS) to 2028 identifies focus themes to support the evaluation of medicines, promote innovation, and ensure patient access to medicines. These areas include accessibility of medicines, leveraging data, digitalization and artificial intelligence, antimicrobial resistance and other health threats, availability and supply of medicines, sustainability of the network, and regulatory science, innovation and competitiveness (1). To keep up with the pace of scientific and technological developments and to accelerate the translation of innovation into medicines, anticipating the need for regulatory support in relation to emerging therapies is of extreme importance (2). To support the future-proofing of the EU innovation ecosystem, it is necessary to monitor and support the integration of advances in science and technology in medicines development and manufacturing (1). With this purpose, EMA uses horizon scanning, which is a foresight tool that applies a set of systematic methods to monitor multiple information sources and identify emerging trends that may impact the future (3). Horizon scanning can be complemented by forecasting approaches, which draw on historical data and trend analysis to inform future outcomes. Together, these approaches support strategic planning and capacity building, strengthen organizational resilience and readiness, and inform regulatory decision-making and the management of risks (4).

## EMA’s approach to horizon scanning

At EMA, the goal of horizon scanning is to identify breakthrough therapies, technological enablers and societal changes expected to impact the development, regulation and use of medicines over a 3-10-year horizon. The EU Innovation Network (EU-IN) is a collaborative initiative that focuses on supporting and coordinating innovation-related activities across authorities responsible for regulating medicines in the European Union. Through the EU-IN, EMA collaborates with NCAs to advance horizon scanning, alongside other innovation-supporting activities (2, 5). This coordinated approach is designed to enable the European medicines regulatory network to respond effectively to emerging pharmaceutical innovation, facilitating timely patient access to innovative medicines and associated technologies while addressing development, regulatory, and procedural bottlenecks. These efforts also strengthen regulatory preparedness and resilience, inform strategic planning, and support agile and adaptive regulatory systems. A flexible yet systematic methodology is essential to maximize the impact of horizon scanning outputs. While individual organizations adapt workflows to their specific mandates, there is broad consensus on the importance of transparent, flexible and context-specific methodologies for signal identification, prioritization and assessment.

At EMA, horizon scanning is implemented through a structured, multi-step process encompassing signal identification, prioritization and assessment (6, 7). To have a broader overview of what is emerging in the field of medicinal product development, a wide variety of information sources such as scientific literature, research and innovation funding calls, grey literature from regulatory authorities and organizations, and publicly available reports are screened daily. The information items are scanned and gathered using an internal collaborative tool, developed at EMA, specifically for this purpose, that supports the daily screening. This evidence is complemented by insights derived from structured stakeholder interactions such as Innovation Task Force (ITF) (8) meetings and Portfolio and Technologies Meetings (PTMs) (9) offered by EMA, which support the identification and prioritization of emerging topics for further assessment. On an annual basis, a signal prioritization exercise is undertaken to identify topics warranting deeper analysis, drawing on expert deliberations and consultations conducted through focused meetings or surveys. Prioritized topics are subsequently assessed through comprehensive analyses of the research and clinical development landscape, ongoing and planned funding activities, applicable regulatory frameworks, scientific and regulatory challenges, and research needs in regulatory science. The outcomes of these assessments are communicated through deep-dive reports, webinars, and follow-up engagements.

## Deep-dive reports: monitoring, assessment, and actions on emerging trends in medicines development

Topic-specific deep-dive reports provide in-depth analyses of emerging areas in medicines development by examining the research landscape, identifying regulatory and scientific challenges, and formulating actionable recommendations for both regulators and innovators (5).

Over the past 2 years, prioritized topics assessed by EMA, in collaboration with the EU-IN, have resulted in deep-dive reports on Alzheimer’s disease (10, 11), nanotechnology-based medicines (12), new approach methodologies (13, 14), artificial intelligence (AI) across the medicines lifecycle (15) and engineered living materials (ELMs) (16). Earlier reports addressed challenges related to fecal microbiota transplantation (17) and genome editing (18).

These reports inform and prompt a range of regulatory actions, including the promotion of early and structured dialogue with developers through EMA ITF meetings or scientific advice procedures, and the identification and development of new or revised guidance by regulators (10, 13). For example, the deep-dive report on Alzheimer’s disease (10) unveiled that there was an incomplete understanding of the disease mechanisms, that animal models failed to translate outcomes, that there were no biomarkers qualified as surrogate endpoints, and highlighted the ethical concerns of diagnosing or treating asymptomatic individuals. These emerging challenges directly informed the development of a concept paper proposing an update of the guideline on the clinical investigation of Alzheimer’s therapies (19).

In addition, these assessments often initiate further targeted activities, such as the organization of multi-stakeholder workshops to discuss and address identified challenges. For instance, the European Innovation Council (EIC)-EMA workshop on the regulatory framework of ELMs, held in early 2025, provided a platform to discuss regulatory challenges and frameworks for these novel products. Insights from this workshop informed the subsequent deep-dive report on ELMs for *in situ* production of therapeutics (16).

Beyond topic-specific analyses, the continuous monitoring and assessment of emerging trends functions as an early foresight mechanism, informing strategic planning and priority-setting across the European medicines regulatory network. Outputs from horizon scanning activities contribute to the network’s strategy (1), to the identification of regulatory science research priorities and funding needs (20), and they support other activities like the AI Observatory published on the EMA website-an initiative designed to monitor, analyze, and coordinate regulatory activities on the use of AI across the medicines lifecycle (15).

## Trends under the regulatory radar

Analysis of third-party reports on emerging trends, produced by public health organizations engaged in horizon scanning, is of high relevance as it helps avoid duplication of effort and supports coordination. As part of EMA’s horizon scanning process, emerging trends identified by these organizations on topics relevant to the European medicines network activities, are systematically collected and integrated into the review. Their relevance is assessed based on unmet medical needs, foreseeable regulatory impact, alignment with the network’s priorities and strategic objectives, as well as the innovative profile of technologies or medicines, including their potential to pose regulatory challenges, reveal knowledge gaps, present public health risks or generate research needs.

Although organizations conducting horizon scanning tailor the scope and depth of their assessments to their specific interests, certain recurring themes become evident. These include gene and cell therapies, encompassing *in vivo* genome editing (21), mRNA vaccines used as therapies (22), chimeric antigen receptor T-cell (CAR-T) therapies (21–23), xenotransplantation (22, 24, 25), and emerging organoid-based biobots (26). Epigenome editing has emerged as an innovative technology offering durable yet reversible interventions; however, challenges related to delivery, off-target effects and ethical issues remain (21). Non-viral gene delivery approaches such as lipid nanoparticles present promising alternatives to enable precise gene therapy, targeted RNA therapy and enhanced mRNA efficacy (21). Advances in bioprinting, including 3D, 4D and *in situ* techniques, as well as the use of microgravity bioreactors are also key areas of technological progress (24, 25, 27, 28) that become relevant to EMA, for example, when they are applied to the manufacturing of Advanced Therapy Medicinal Products (ATMPs). While bioprinting is widely applied in tissue regeneration, 3D printed scaffolds can also be used for controlled drug release applications (21). Innovative gene and cell therapies, and delivery technologies offer opportunities but raise regulatory concerns about product classification, scalable manufacturing, quality assurance, and post-marketing safety monitoring (29).

Microbiome engineering is emerging as a transformative area for personalized medicines (17, 30), while innovation in drug delivery, such as the use of extracellular vesicles (EVs) is also gaining ground (21). Engineered EVs have the potential to deliver proteins, RNA and medicines directly to target tissues and are being studied across oncology, neurology and regenerative therapies. Advances in EVs targeting through *in situ* production and modulation are expected to further enhance their therapeutic potential (21). Nonetheless, issues with large-scale production, reproducibility, and regulatory standards still hinder clinical use (31).

Glucagon-like peptide-1 (GLP-1) receptor agonists, widely used to treat diabetes and weight management, are being investigated for potential new therapeutic indications, including possible neuroprotective effects (32). Consideration must be given to the implications for regulatory evaluation, availability, and evidence requirements, including benefit-risk reassessment and possible label extension (32).

Innovative technologies can enable the translation of innovation into novel medicinal products. Therefore, it is crucial to stay informed about developments in medical technology. For EMA, innovative tools, methods, or approaches that play a major role in shaping and speeding up the development of medicines, are considered enabling technologies.

Neurotechnology represents a major emerging trend, encompassing for example, brain-machine interfaces (BMIs) used to decode human thoughts (33), therapeutic deep brain stimulation closed loop devices to treat neurological conditions (34), and neuroimaging techniques that can enable the development of novel biomarkers (33). These technologies have the potential to improve early detection, treatment and management of serious neurological diseases (33, 34). However, issues of neural data ownership and device safety may hinder their adoption (30, 33–35). Additional speculative ethical concerns arise from the potential use of these devices to edit memories (34).

AI’s use in healthcare and medicines development remains a central focus, particularly the rise of generative AI, human-like AI systems, and the application of digital twins in clinical trials (15, 36, 37). Digital twins are used to simulate, predict and optimize outcomes across the medicines lifecycle, enabling the modelling of personalized treatment strategies, the monitoring and prediction of health status, and the exploration of the impact of early interventions (35). The combination of AI with digital biomarkers is particularly promising as advanced algorithms can integrate and analyze complex datasets, including genomic information, electronic health records and data from wearable devices to enhance predictive accuracy (25, 33, 36). AI tools and applications have the potential to transform the way medicines are developed and evaluated. The fast pace of AI developments is a challenge for both developers and regulatory authorities (37). When using AI for evidence generation across all phases of medicines lifecycle, the technologies should be fit for purpose and align with legal, ethical, technical and scientific standards established by EU legislation (38) and applicable EMA guidance (39, 40).

EMA integrates these emerging trends that could affect medicinal product development and regulatory evaluation into its signal scanning, combining internal regulatory intelligence with peer-reviewed literature and external foresight reports.

## Collaborations and international outreach

As multiple organizations undertake horizon scanning, using different methodologies and sometimes addressing common topics, efforts are underway to strengthen collaboration, promote knowledge sharing and encourage joint activities. In this context, EMA organized a webinar on 20 May 2025 dedicated to horizon scanning methodologies and emerging topics of interest. The event provided a forum for dialogue, facilitated the exchange of experiences, and highlighted opportunities and challenges, thereby fostering closer interaction among experts in the field. Organizations actively engaged in health-related horizon scanning include the European Commission Joint Research Centre (EC-JRC), the World Health Organization (WHO), the European Food Safety Authority (EFSA), and the National Institute for Health and Care Research Innovation Observatory (NIHR IO). The event facilitated ongoing knowledge exchange and collaboration among the participating institutions (1).

Beyond this initiative, EMA collaborates in a range of ongoing actions aimed at strengthening foresight across EU institutions. These activities include the mapping and sharing of existing foresight methodologies and outputs across agencies, as exemplified by the EU Agencies Network on Scientific Advice (ANSA) Futures Cluster’s comprehensive report on the foresight practices (41). Agencies participating in the EU-ANSA Futures Cluster are also collaborating to identify and pursue shared topics of interest, including the development of joint foresight initiatives (41). In parallel, regular exchanges with other competent authorities, such as Swissmedic, have been established to support international alignment and information exchange.

To ensure sustained cooperation and knowledge exchange, efforts are underway to establish dedicated working groups and regular fora at the European level. One example is the emerging foresight community of practice for EU foresight practitioners, led by the EU Policy Lab within the JRC’s Competence Centre on Foresight.

Collectively, these initiatives contribute to embedding foresight within regulatory and policy processes, support evidence-informed responses, and help ensure that regulatory and research agendas remain aligned with the rapidly evolving landscape of health and medicines innovation. Collaboration supports the goals of the EMANS to 2028, which aims to enhance regulatory science, foster innovation and competitiveness, future-proof the EU innovation ecosystem, and implement a coordinated EU horizon scanning for medicines (1).

## Discussion

At EMA, horizon scanning underpins a proactive, forward-looking approach that supports the objectives of the EMANS 2028 in regulatory science, innovation and competitiveness. It assists in determining essential training requirements for the network and ultimately supports the identification of expertise needed for future product evaluation. This process promotes cooperation with NCAs to accelerate the translation of innovation into therapies and thus increasing EU competitiveness. By fostering such partnerships, regulators can ensure that their teams are equipped with the up-to-date knowledge and skills required to address new challenges. Horizon scanning supports regulatory preparedness and resilience by enabling foresight of emerging innovations in medicines development and technologies. It allows regulators to adapt regulatory frameworks, capacities, research priorities, and strategies in a timely manner. This ensures that regulatory bodies across EU can remain responsive to new trends and challenges, ultimately supporting the development of innovative, safe, and effective medicines for patients.

## Data Availability

The original contributions presented in the study are included in the article/supplementary material, further inquiries can be directed to the corresponding author.

## Author’s note

The views expressed in this article are the personal views of the authors and may not be understood or quoted as being made on behalf of or reflecting the position of the European Medicines Agency or one of its committees or working parties.
